# Comprehensive evaluation of the prediction of fetal growth restriction using the ratio of fetal cerebellar transverse diameter to abdominal circumference combined with uterine artery blood flow parameters

**DOI:** 10.3389/fped.2025.1633122

**Published:** 2025-07-23

**Authors:** Weiying Tang, Pingping Wu, Yanyan Zheng, Baobao Yang

**Affiliations:** ^1^ Department of Obstetrics, Cangnan People ’s Hospital, Wenzhou, Zhejiang, China; ^2^ Department of Ultrasound, Cangnan People ’s Hospital, Wenzhou, Zhejiang, China

**Keywords:** fetal growth restriction, ratio of fetal cerebellar transverse diameter to abdominal circumference, uterine artery blood flow parameters, prediction value, ultrasound examination, blood biomarkers

## Abstract

**Objective:**

To explore the value of the ratio of fetal cerebellar transverse diameter to abdominal circumference (TCD/AC) combined with uterine artery blood flow parameters in the assessment of fetal growth restriction (FGR).

**Methods:**

A retrospective analysis was conducted, including 152 women diagnosed with FGR through prenatal ultrasound screening at our hospital between January 2020 and December 2024 as the FGR group, and 156 pregnant women with normal prenatal examinations during the same period were included as the non-FGR group using a stratified sampling method. Parameters such as TCD/AC, head circumference to abdominal circumference ratio (HC/AC), and hemodynamic indicators of uterine and cerebral artery blood flow were measured through ultrasound examinations. Blood biomarkers such as insulin-like growth factor 1 (IGF-1), placental growth factor (PlGF), and vascular endothelial growth factor (VEGF) were also assessed.

**Results:**

There were no statistically significant differences between the two groups in terms of age, BMI, gestational weeks, parity, and gravidity (*P* > 0.05). The TCD/AC and HC/AC ratios in the FGR group were significantly lower than those in the non-FGR group (*P* < 0.05), while the uterine artery pulsatility index (PI), resistance index (RI), and systolic to diastolic peak velocity ratio (S/D) were significantly higher in the FGR group (*P* < 0.05). Additionally, levels of IGF-1, PlGF, and VEGF were significantly lower in the FGR group (*P* < 0.05). Multivariable logistic regression analysis revealed that TCD/AC, uterine artery PI (UtA-PI), uterine artery RI (UtA-RI), and uterine artery S/D (UtA-S/D) were independent predictors of FGR. Receiver operating characteristic (ROC) curve analysis demonstrated that when these indicators were used in combination, the diagnostic efficiency of FGR was improved, with an AUC of 0.820.

**Conclusion:**

The combination of TCD/AC with uterine artery blood flow parameters has high predictive value for FGR and can serve as an effective tool for early identification and management of FGR in clinical practice.

## 1 Introduction

Fetal Growth Restriction (FGR), also known as intrauterine growth restriction, refers to a condition where the fetus does not achieve its genetic potential for growth while in the womb (1). It is commonly defined as a birth weight below two standard deviations of the mean for the gestational age or below the 10th percentile of normal weight for the same age (2). In China, the incidence of FGR is reported to be 6.39%, making it the second leading cause of perinatal mortality, with a mortality rate 6–10 times higher than that of normally developing infants (3). The causes of FGR are diverse and involve factors related to the fetus, the mother, the umbilical cord, and the placenta. Chromosomal or genetic abnormalities in the fetus and deficiencies in key growth-regulating substances such as leptin and growth hormone can lead to FGR. Maternal risk factors include severe pregnancy complications, malnutrition, pregnancy-related disorders, advanced maternal age, smoking, and alcohol consumption (4). Additionally, factors such as a thin or long umbilical cord, knots or twists in the cord, and reduced blood flow due to placental abnormalities are significant contributors to FGR. FGR not only affects fetal growth and development but can also result in serious consequences such as neonatal hypoglycemia, delayed intellectual development, and even intrauterine death (5).

The Transcerebellar Diameter to Abdominal Circumference Ratio (TCD/AC) is an indicator used to assess the relative size of the fetal head to the abdomen, aiding in determining the correct gestational age and reflecting the development of the fetal cerebellum (6). Abdominal circumference, on the other hand, is used to evaluate the overall size and growth status of the fetus. Uterine artery blood flow parameters are crucial indicators for monitoring uterine blood perfusion, reflecting the placental blood flow within the uterus, and thereby assessing fetal health and potential pregnancy complications (7). These parameters are typically obtained through ultrasound examinations and serve as effective tools for evaluating fetal health for clinicians (8).

Given the individual importance of TCD/AC and uterine artery blood flow parameters in assessing fetal growth, this study aims to investigate the value of using these two indicators in combination for predicting FGR. Through a retrospective analysis, we hope to provide a more comprehensive and reliable screening method for FGR in clinical practice, enhancing early identification and intervention capabilities for this high-risk pregnancy condition, and offering valuable insights for clinical decision-making.

## 2 Materials and methods

### 2.1 Subject selection

This study employed a retrospective analysis, including 152 women diagnosed with fetal growth restriction (FGR) through prenatal ultrasound screening at our hospital between January 2020 and December 2024 as the FGR group, and 156 pregnant women with normal prenatal examinations during the same period were included as the non-FGR group using a stratified sampling method. The gestational weeks of pregnant women in FGR group correspond one-to-one with those in the non-FGR group.

Inclusion criteria for FGR group:

(1) Clinical diagnosis consistent with patients with fetal growth restriction (9), birth weight < 2 standard deviations of the mean weight for the corresponding gestational age;
(2) Singleton pregnancy;
(3) Natural pregnancy;
(4) Regular menstrual cycles, with clear last menstrual period;
(5) Pregnant women have no complications or comorbidities during pregnancy.

Inclusion criteria for non-FGR group:

(1) Singleton pregnancy;
(2) Natural pregnancy;
(3) Regular menstrual cycles, with clear last menstrual period;
(4) No obvious fetal abnormalities were found during fetal screening, and fetal development was consistent with gestational age;
(5) Pregnant women have no complications or comorbidities during pregnancy;
(6) Follow up of fetal weight after birth to exclude FGR.

Exclusion Criteria:

(1) Abnormal fetal status detected during maternal check-ups;
(2) Maternal substance abuse, alcoholism, or mental disorders;
(3) Maternal intrauterine infection;
(4) Occurrence of other adverse outcomes during pregnancy;
(5) Incomplete clinical data.

This study has obtained approval from the Cangnan People 's Hospital Institutional Review Board (IRB). The research protocol adheres to the principles outlined in the Helsinki Declaration. Since this retrospective study only utilized de-identified patient data without potential harm or impact on patient care, the requirement for informed consent was waived by our hospital's institutional review board and ethics committee.

### 2.2 Instruments and methods

#### 2.2.1 Instruments

The Voluson E8 color Doppler ultrasound diagnostic instrument manufactured by GE Healthcare in the United States was utilized. The probe frequency ranged from 2.5 to 5 MHz. It features functions such as cine playback, local zoom, and tissue harmonic imaging. All ultrasound examinations were conducted by two experienced sonographers to ensure the consistency and accuracy of the data.

#### 2.2.2 Ultrasound examination

**Figure 1:**
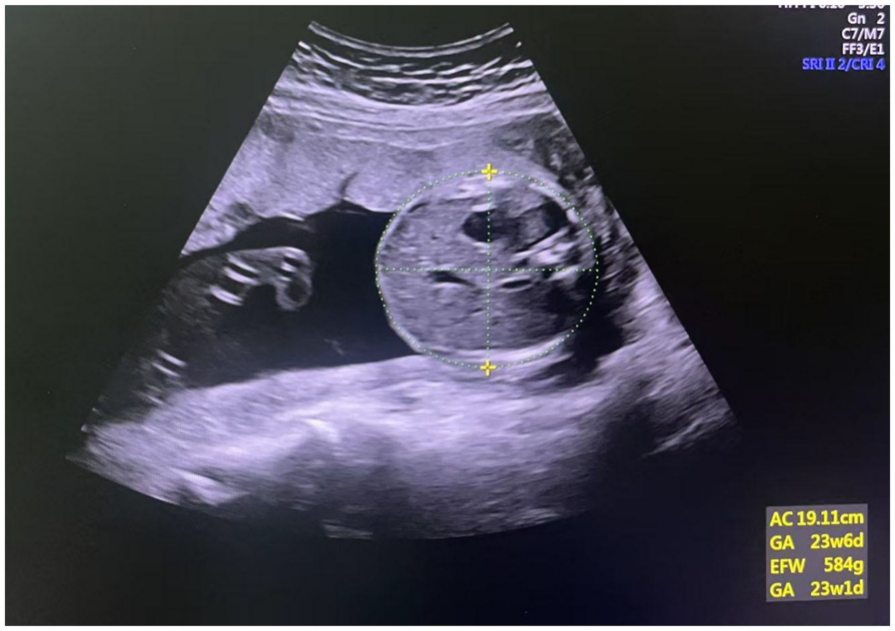
Measurements of the abdominal circumference.

During the ultrasound examination (gestational weeks 20–24), pregnant women were positioned in a semi-recumbent posture with a slight left tilt to reduce the risk of supine hypotension syndrome caused by compression of the inferior vena cava. Body positioning was adjusted as needed to obtain optimal measurement angles. Routine fetal growth parameters, including biparietal diameter (BPD), head circumference (HC), abdominal circumference (AC), and femur length (FL), were measured using a 3.5 MHz abdominal probe. Estimated fetal weight was calculated using the built-in software of the GE Voluson E8 instrument. The AC measurement is shown in Figure 1.

**Figure 2:**
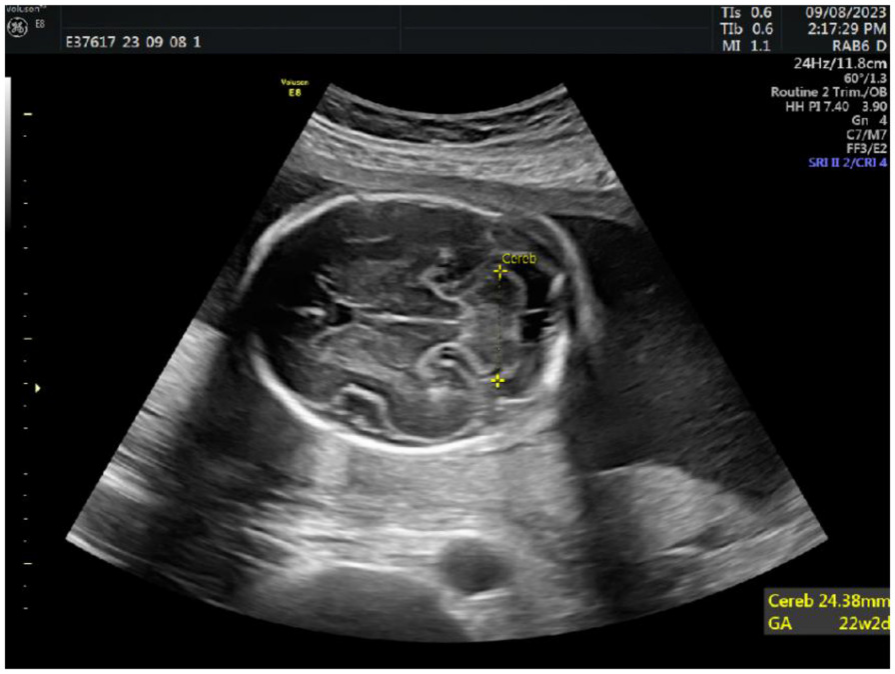
Measurements of the transcerebellar diameter.

Additionally, detailed assessments of fetal cranial structures, thoracoabdominal regions, heart, and limbs were performed. In the horizontal section of the fetal head, the cerebellum, cisterna magna, and cavum septi pellucidi were clearly visualized, typically including bilateral anterior horns of the lateral ventricles. Measurements of the transcerebellar diameter were taken by placing the calipers at the outer edge of the cerebellum for precise values, see Figure 2.

**Figure 3:**
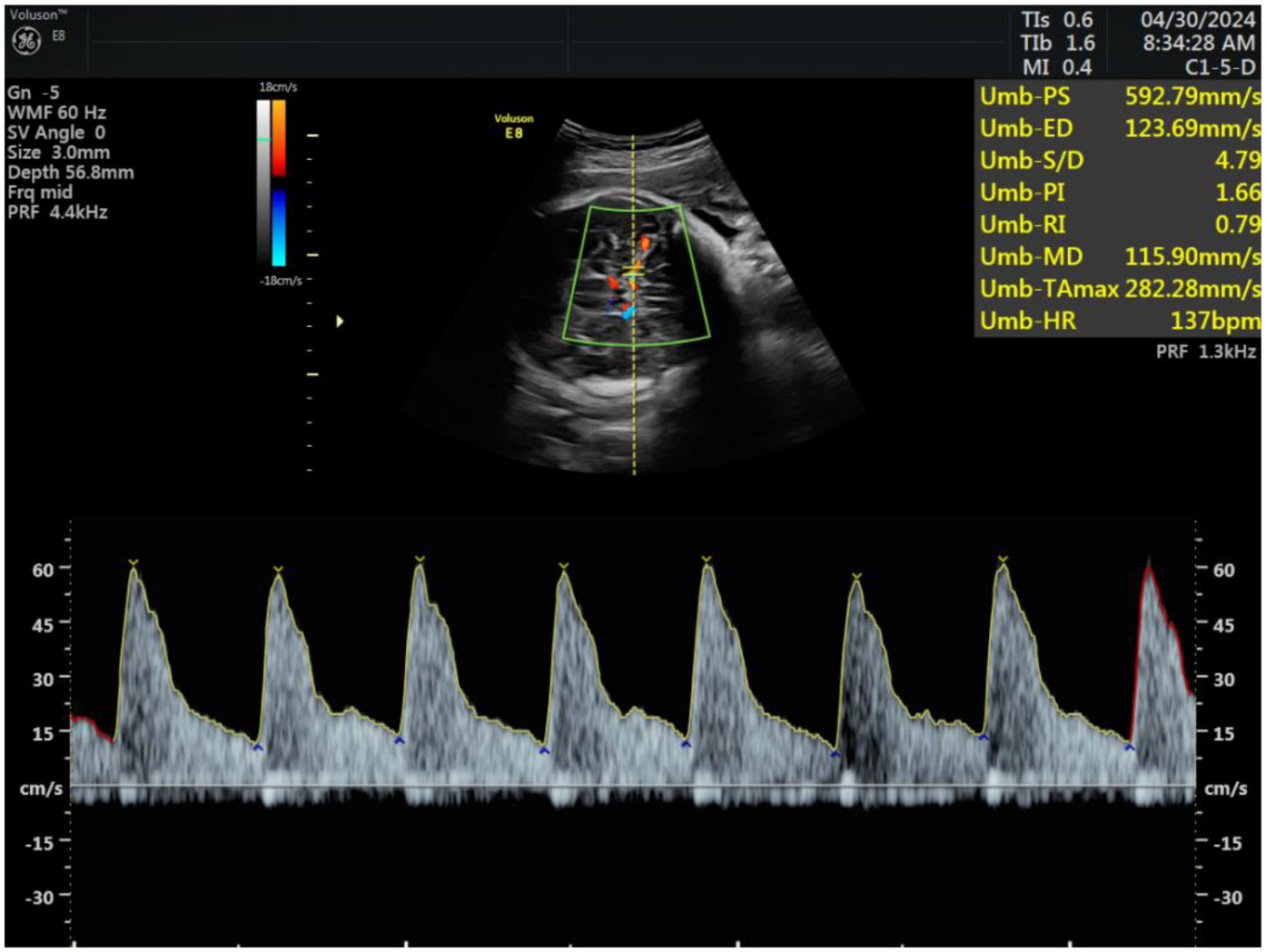
Measurement of fetal middle cerebral artery and normal waveform.

Subsequently, hemodynamic parameters of the fetal middle cerebral artery and maternal uterine artery blood flow were measured. Doppler flow imaging was used to locate the uterine artery at the intersection with the external iliac artery in the lower abdomen. Samples were taken approximately 1 cm from the intersection point, ensuring that the blood flow sampling line was parallel to the direction of the uterine artery and the angle between the beam and the flow did not exceed 15°. The pulsatility index (PI), resistance index (RI), and systolic-to-diastolic peak velocity ratio (S/D) of the uterine artery were recorded and averaged after three repeated measurements. Similarly, Doppler flow imaging of the fetal middle cerebral artery was performed, and measurements of PI, RI, and S/D were taken, with the angle between the beam and the flow did not exceed 15°, repeating each measurement three times and averaging the results, see Figure 3.

### 2.3 Blood tests

Blood samples collected from all study subjects during late pregnancy (gestational weeks 28 to 34) were analyzed. These samples were collected during routine prenatal examinations and processed according to standard operating procedures. Pregnant women were instructed to fast for at least 8 h before blood collection to ensure the accuracy of the results. Blood samples were obtained via standard venipuncture techniques from the antecubital or dorsal hand veins, with approximately 8 ml of whole blood collected per individual. Of this, 4 ml was used for serum separation (centrifuged at 3,000 rpm for 10 min to obtain the supernatant) and 4 ml for plasma separation. Blood samples were immediately placed in tubes containing anticoagulants (such as EDTA) and processed promptly to avoid factors that could affect the analysis, such as hemolysis. Levels of tumor necrosis factor-alpha (TNF-α), insulin-like growth factor 1 (IGF-1), placental growth factor (PlGF), and vascular endothelial growth factor (VEGF) were measured using enzyme-linked immunosorbent assay (ELISA) kits from the Jiangsu Jiancheng Bioengineering Institute in China. Concentrations of alpha-fetoprotein (AFP), beta-human chorionic gonadotropin (β-hCG), and unconjugated estriol (uE3) were determined using electrochemiluminescence immunoassay (ECLIA) and quantitative analysis with reagent kits from Roche Diagnostics Limited. Key parameters reflecting maternal coagulation function, including activated partial thromboplastin time (APTT) and D-dimer, were assessed. APTT measurements were conducted using a fully automated coagulation analyzer following the manufacturer's standard operating procedures with the CA-7000 series instrument from Sysmex Corporation in Japan. D-dimer detection was performed using an immunoturbidimetric assay with kits from the Jiangsu Jiancheng Bioengineering Institute in China. All tests adhered to standard laboratory operating procedures (SOPs) and underwent regular internal and external quality control checks to ensure the accuracy and reliability of the results.

#### 2.3.1 Observational indicators

(1) General information: including age, BMI, gestational age, gravidity, and parity;
(2) Ultrasound findings: including transcerebellar diameter to abdominal circumference ratio (TCD/AC) and head circumference to abdominal circumference ratio (HC/AC);
(3) Hemodynamic parameters of uterine and fetal middle cerebral artery blood flow: including uterine artery pulsatility index (UtA-PI), resistance index (UtA-RI), and systolic-to-diastolic peak velocity ratio (UtA-S/D); fetal middle cerebral artery pulsatility index (MCA-PI), resistance index (MCA-RI), and systolic-to-diastolic peak velocity ratio (MCA-S/D);
(4) Blood biomarkers: including TNF-α, IGF-1, PlGF, VEGF, AFP, β-hCG, uE3, etc;
(5) Coagulation function indicators: including APTT, D-dimer;
(6) Down syndrome screening markers: including AFP, β-hCG, and uE3;
(7) Multifactorial logistic regression analysis: identifying independent risk factors for FGR;
(8) ROC curve analysis: evaluating the diagnostic value of TCD/AC ratio combined with uterine artery blood flow parameters for FGR;
(9) Neonatal outcomes: including premature birth rate, neonatal weight, amniotic index, and 1-min Apgar score.

### 2.4 Statistical analysis

Data analysis was performed using SPSS 22.0 software (IBM Corp., Armonk, NY, USA). Quantitative data were presented as mean ± standard deviation, and comparisons between groups were conducted using independent-sample *t*-tests or analysis of variance (ANOVA). Qualitative data were expressed as frequencies (percentages), and group differences were assessed using the chi-squared test. Spearman correlation analysis was used to explore the relationships between variables and FGR. Multifactorial logistic regression models were employed to analyze independent risk factors for FGR. ROC curves were plotted, and the area under the curve (AUC) was calculated to evaluate the predictive value of the TCD/AC ratio combined with uterine artery blood flow parameters for FGR, with statistical significance set at *P* < 0.05.

## 3 Results

### 3.1 Analysis of differences in participant characteristics between two groups

There were no statistically significant differences between the two groups in terms of age, BMI, gestational age, gravidity, and parity (*P* > 0.05), indicating that these general characteristics were well-matched between the non-FGR group and the FGR group, as shown in Table 1.

**Table 1:** Univariate analysis of factors influencing fetal growth restriction.

Parameters	Non-FGR group (*n* = 156)	FGR group (*n* = 152)	*t*/*χ*2	*P*
Age (years)	28.57 ± 3.08	28.08 ± 3.00	1.423	0.156
BMI (kg/m2)	23.66 ± 3.22	23.35 ± 3.29	0.834	0.405
Gestational age (weeks)	36.91 ± 1.96	36.90 ± 2.19	0.065	0.948
Gravidity [*n* (%)]			0.153	0.696
Primary	70 (44.87%)	64 (42.11%)		
Multiple	88 (56.41%)	88 (57.89%)		
Parity [*n* (%)]			0.004	0.947
Primary	95 (60.90%)	92 (60.53%)		
Multiple	61 (39.10%)	60 (39.47%)		

BMI, body mass index.

### 3.2 Comparison of ultrasound examination results

The ratio of fetal cerebellar transverse diameter to abdominal circumference (TCD/AC) and the ratio of head circumference to abdominal circumference (HC/AC) were significantly lower in the FGR group compared to the non-FGR group (*P* < 0.05), as detailed in Table 2.

**Table 2:** Analysis of differences in ultrasound examination results.

Parameters	Non-FGR group (*n* = 156)	FGR group (*n* = 152)	*t*	*P*
TCD/AC	1.89 ± 0.43	1.72 ± 0.45	3.222	0.001
HC/AC	1.13 ± 0.04	1.09 ± 0.05	7.211	<0.001

TCD/AC: Ratio of fetal cerebellar transverse diameter to abdominal circumference; HC/AC: Ratio of head circumference to abdominal circumference.

### 3.3 Comparison of hemodynamic parameters in uterine artery and middle cerebral artery

Hemodynamic parameters in the uterine artery such as PI, RI, and S/D were significantly higher in the FGR group compared to the non-FGR group (*P* < 0.05). In contrast, the PI, RI, and S/D of the middle cerebral artery were significantly lower in the FGR group compared to the non-FGR group (*P* < 0.05), as shown in Table 3.

**Table 3:** Analysis of differences in hemodynamic parameters of uterine artery and middle cerebral artery.

Parameters	Non-FGR group (*n* = 156)	FGR group (*n* = 152)	*t*	*P*
UtA-PI	1.72 ± 0.21	1.87 ± 0.33	4.817	<0.001
UtA-RI	0.74 ± 0.24	0.82 ± 0.33	2.420	0.016
UtA-S/D	2.47 ± 0.64	2.91 ± 0.59	6.350	<0.001
MCA-PI	1.76 ± 0.55	1.55 ± 0.38	3.976	<0.001
MCA-RI	0.79 ± 0.21	0.72 ± 0.11	3.556	<0.001
MCA-S/D	3.44 ± 0.92	3.09 ± 0.85	3.496	0.001

UtA-PI, uterine artery pulsatility index; UtA-RI, uterine artery resistance index; UtA-S/D, uterine artery systolic to diastolic flow velocity ratio; MCA-PI, middle cerebral artery pulsatility index; MCA-RI, middle cerebral artery resistance index; MCA-S/D, middle cerebral artery systolic to diastolic flow velocity ratio.

### 3.4 Comparison of inflammatory markers, coagulation function, and growth factors

In terms of inflammatory markers, coagulation function, and growth factors, IGF-1, PlGF, and VEGF were significantly lower in the FGR group compared to the non-FGR group (*P* < 0.05). However, other indicators including TNF-α, neutrophil count, white blood cell count (WBC), APTT, D-dimer, and CRP showed no significant differences between the two groups (*P* > 0.05), as depicted in Table 4.

**Table 4:** Analysis of differences in inflammatory markers, coagulation function, and growth factors.

Parameters	Non-FGR group (*n* = 156)	FGR group (*n* = 152)	*t*	*P*
TNF-α (pg/ml)	1.93 ± 0.49	1.95 ± 0.52	0.303	0.762
Neutrophil (×10^9/L)	2.09 ± 0.76	2.10 ± 0.85	0.166	0.868
WBC (×10^9/L)	7.46 ± 1.49	7.45 ± 1.56	0.009	0.993
APTT (s)	35.20 ± 4.83	35.40 ± 3.68	0.402	0.688
D-dimer (mg/L)	0.47 ± 0.15	0.49 ± 0.16	1.184	0.237
CRP (mg/L)	4.95 ± 1.26	5.18 ± 1.18	1.671	0.096
IGF-1 (ng/ml)	158.40 ± 33.79	133.95 ± 30.93	6.618	< 0.001
PlGF (pg/ml)	135.50 ± 28.04	115.34 ± 22.99	6.890	< 0.001
VEGF (pg/ml)	112.02 ± 22.28	94.21 ± 18.49	7.623	< 0.001

TNF-α, tumor necrosis factor alpha; APTT, activated partial thromboplastin time; D-dimer, D-dimer; CRP, C-reactive protein; IGF-1, insulin-like growth factor 1; PlGF, placental growth factor; VEGF, vascular endothelial growth factor.

### 3.5 Comparison of down syndrome screening indicators in two groups of pregnant women

Comparison of Down syndrome screening indicators revealed that alpha-fetoprotein (AFP), beta-human chorionic gonadotropin (β-hCG), and unconjugated estriol (uE3) were significantly different in the FGR group compared to the non-FGR group (*P* < 0.05), as shown in Table 5.

**Table 5:** Comparison of down syndrome screening indicators in Two groups of pregnant women.

Groups	*n*	AFP (ng/ml)	β-hCG (U/L)	uE3 (ng/ml)
FGR group	152	55.48 ± 7.75	5.84 ± 1.72	1.17 ± 0.47
Non-FGR group	156	48.63 ± 4.15	4.54 ± 1.09	1.53 ± 0.29
*t*		9.711	7.926	8.140
*P*		< 0.001	< 0.001	< 0.001

AFP, alpha-fetoprotein; β-hCG, beta-human chorionic gonadotropin; uE3, unconjugated estriol.

### 3.6 Correlation analysis

The correlation analysis revealed significant associations between FGR and multiple variables. The TCD/AC ratio, HC/AC ratio, IGF-1, PlGF, VEGF, uE3 levels, neonatal body weight, amniotic fluid index, and 1-minute Apgar score were negatively correlated with FGR (*P* < 0.05), while uterine artery blood flow parameters UtA-PI, UtA-RI, UtA-S/D, as well as AFP, β-hCG levels, and the rate of preterm birth were positively correlated with FGR (*P* < 0.05), as shown in Table 6.

**Table 6:** Correlation analysis of variables with FGR.

Variables	Correlation coefficients (rho)	*P*-values
β-hCG（U/L）	0.4049	<0.001
VEGF (pg/ml)	−0.3975	<0.001
UtA-S/D	0.3252	<0.001
UtA-RI	0.1279	0.0247
UtA-PI	0.2497	<0.001
uE3（ng/ml）	−0.4244	<0.001
TCD/AC	−0.1765	0.0019
PlGF (pg/ml)	−0.3579	<0.001
MCA-S/D	−0.1844	0.0012
MCA-RI	−0.2022	0.0004
MCA-PI	−0.2283	<0.001
IGF-1 (ng/ml)	-0.3375	<0.001
HC/AC	−0.3845	<0.001
AFP（ng/ml）	0.5119	<0.001

β-hCG, beta-human chorionic gonadotropin; VEGF, vascular endothelial growth factor; UtA-S/D, uterine artery systolic to diastolic flow velocity ratio; UtA-RI, uterine artery resistance index; UtA-PI, uterine artery pulsatility index; uE3, unconjugated estriol; TCD/AC, ratio of fetal cerebellar transverse diameter to abdominal circumference; PlGF, placental growth factor; MCA-S/D, middle cerebral artery systolic to diastolic flow velocity ratio; MCA-RI, middle cerebral artery resistance index; MCA-PI, middle cerebral artery pulsatility index; IGF-1, insulin-like growth factor 1; HC/AC, ratio of head circumference to abdominal circumference; AFP, alpha-fetoprotein.

### 3.7 Multifactor logistic regression analysis of the impact of fetal cerebellar transverse diameter to abdominal circumference ratio combined with uterine artery blood flow parameters on FGR

Using UA-PI, UA-RI, UA-S/D, TCD/AC, HC/AC, β-hCG, uE3, and AFP as independent variables with their respective values, a logistic regression analysis was conducted with the outcome of fetal growth restriction (FGR = 1, no FGR = 0). The results of the multifactor logistic regression analysis indicated that UA-PI, UA-RI, UA-S/D, and TCD/AC were independent influencing factors for fetal growth restriction (*P* < 0.05), as shown in Table 7.

**Table 7:** Factors influencing FGR in multifactor logistic regression analysis.

Risk factor	β	SE	Ward	OR	95%CI	*P*
TCD/AC	−0.945	0.411	5.286	0.384	0.175∼0.892	0.027
UtA-PI	2.933	0.746	15.457	19.107	4.211∼83.402	<0.001
UtA-RI	1.284	0.651	2.157	4.166	1.098∼12.314	0.036
UtA-S/D	1.588	0.325	4.114	4.875	2.403∼10.021	<0.001

### 3.8 ROC curve analysis of the evaluation value of fetal cerebellar transverse diameter to abdominal circumference ratio combined with uterine artery blood flow parameters for FGR

**Figure 4:**
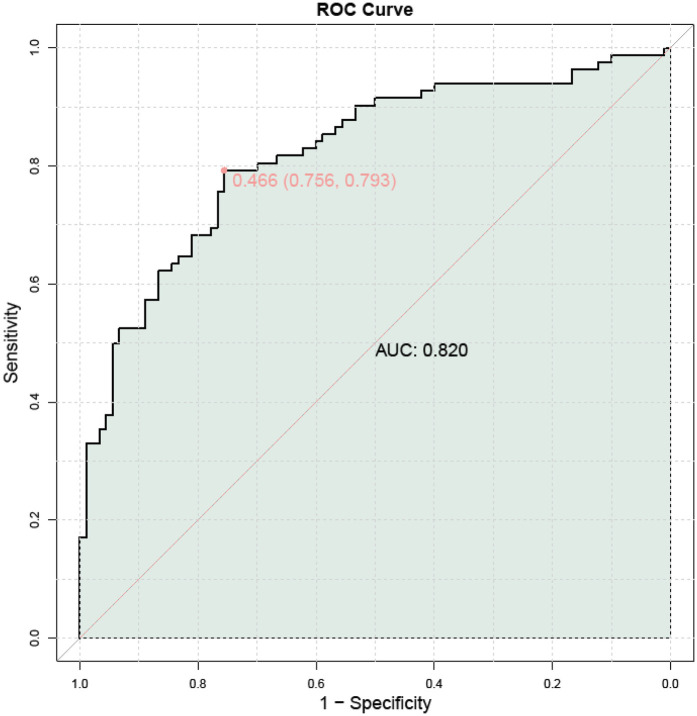
Combined ROC curve analysis of the evaluation value of fetal cerebellar transverse diameter to abdominal circumference ratio combined with uterine artery blood flow parameters for FGR.

ROC curve analysis demonstrated that the combination of TCD/AC with uterine artery blood flow parameters had diagnostic value for assessing FGR. Specifically, the optimal threshold for TCD/AC was 1.715, with a sensitivity of 0.524, specificity of 0.722, and AUC of 0.633. The optimal thresholds for UtA-PI, UtA-RI, and UtA-S/D were also determined. Considering the limited predictive efficacy of individual indicators, a combined ROC curve analysis was performed to explore the potential advantages of using these indicators together. The combined ROC curve analysis indicated that the integration of these indicators could enhance the predictive performance for FGR, with an AUC of 0.820, as shown in Table 8 and Figure 4.

**Table 8:** ROC analysis results.

Parameters	Optimal threshold	Sensitivity	Specificity	AUC	Youden index
TCD/AC	1.755	0.546	0.622	0.602	0.116
UtA-PI	1.885	0.500	0.801	0.644	0.129
UtA-RI	0.975	0.342	0.840	0.574	0.117
UtA-S/D	2.795	0.605	0.692	0.688	0.129

AUC, area under the curve.

### 3.9 Comparison of neonatal outcomes in Two groups

The FGR group had a higher incidence of preterm births compared to the non-FGR group. Additionally, neonatal body weight, amniotic fluid index, and 1-minute Apgar score were lower in the FGR group than in the non-FGR group (*P* < 0.05), as shown in Table 9.

**Table 9:** Comparison of neonatal outcomes in Two groups.

Groups	Case	Preterm (*N*)	Neonatal body weight	Amniotic fluid index	1-min Apgar score
FGR group	152	18	2.07 ± 0.25	10.95 ± 1.60	9.06 ± 1.19
Non-FGR group	156	2	3.69 ± 0.31	12.11 ± 1.87	9.93 ± 0.45
*t*/*χ*^2^		14.139	50.275	5.799	8.569
*P*		<0.001	<0.001	<0.001	<0.001

## 4 Discussion

Through a retrospective analysis, this study explored the assessment value of TCD/AC combined with uterine artery blood flow parameters for FGR. The results of the study indicate that the TCD/AC ratio and uterine artery hemodynamic parameters have significant predictive value for FGR, and the combined use of these parameters can enhance diagnostic efficiency.

The study findings revealed that the TCD/AC ratio was significantly lower in the FGR group compared to the non-FGR group, consistent with previous research (10, 11). TCD/AC serves as a crucial indicator reflecting the relative size of the fetal head to the abdomen, with a decrease suggesting potential fetal growth retardation. Furthermore, the observed decrease in HC/AC in the FGR group further supports this conclusion. These changes in ultrasound measurement parameters may be attributed to fetal malnutrition or placental dysfunction leading to overall fetal growth restriction (6, 12, 13).

Changes in uterine artery blood flow parameters are essential for understanding placental function. Our study results indicated that parameters such as PI, RI, and S/D in uterine artery blood flow were elevated in the FGR group, while corresponding parameters in the middle cerebral artery were decreased. These alterations reflect inadequate uteroplacental circulation, potentially resulting in reduced oxygen and nutrient supply to the fetus, thereby affecting normal fetal growth (14, 15). Notably, the decrease in middle cerebral artery blood flow parameters may indicate the initiation of fetal brain protection mechanisms to ensure prioritized blood supply to the brain (16).

The multifactor logistic regression model confirmed that TCD/AC and uterine artery blood flow parameters were independent predictive factors for FGR. This suggests that even after adjusting for other potential confounding factors, these ultrasound measurement parameters can still provide crucial information about FGR, serving as a basis for early intervention. The ROC curve analysis further supported this, indicating that the diagnostic efficiency is superior when these parameters are used in combination, suggesting that considering multiple biomarkers can enhance the accuracy of FGR screening, providing a new perspective for FGR screening in clinical practice.

IGF-1, PlGF, and VEGF levels were significantly lower in the FGR group compared to the non-FGR group, while inflammatory markers such as TNF-α showed no significant differences. This suggests that the reduction in growth factors may be a significant mechanism in FGR, with inflammation playing a secondary role in this process (17–19). These findings aid in better understanding the molecular basis of FGR occurrence and provide clues for future treatment strategies. Detecting the concentrations of AFP, β - HCG, and μ E3 in maternal serum can to some extent reflect fetal growth and development, but cannot comprehensively evaluate the various possible causes and specific conditions of FGR. There were significant differences in AFP, β - hCG, and uE3 levels between the FGR group and the non FGR group, indicating abnormal placental function in FGR. Correlation analysis also showed a negative correlation between uE3 levels and FGR. AFP and β - hCG levels were positively correlated with FGR. The significant variation in Down syndrome screening indicators may be related to FGR.

Neonates in the FGR group exhibited higher rates of preterm birth, lower birth weights, amniotic fluid index, and 1-minute Apgar scores, aligning with existing literature reports (20–22). These adverse outcomes underscore the importance of early identification and management of FGR to improve neonatal health. Additionally, they highlight the need for enhanced prenatal care and postnatal support to mitigate the long-term effects of FGR.

The results of this study indicate that the combined use of TCD/AC and uterine artery blood flow parameters can serve as an effective predictive tool for FGR. However, further validation of these findings through larger prospective studies is necessary to determine the optimal thresholds and clinical utility of these indicators. Additionally, exploring more potential biomarkers is essential to enhance the diagnostic accuracy and preventive effects of FGR.

While this study yielded promising results, some limitations exist. Firstly, the retrospective design of the study may introduce selection bias. Secondly, the relatively small sample size may impact the generalizability of the results. Furthermore, despite considering various factors, not all potential influences on FGR, such as genetic background, were covered. Meanwhile, the ultrasound examination results depend on the operator's personal technical level, which may affect the deviation of the results. Finally, as the study subjects were from a single center, further studies are needed to validate our findings in more centers and a wider population.

## 5 Conclusion

In conclusion, the combined use of TCD/AC with uterine artery blood flow parameters demonstrates high value in predicting fetal growth restriction. These parameters not only aid physicians in more accurately assessing fetal growth status but also enable the early detection of potential risks, providing robust support for clinical decision-making. Therefore, it is recommended to further apply and promote these combined predictive indicators in clinical practice to better safeguard maternal and infant health, reduce the incidence of perinatal complications, and enhance the quality of life for newborns. In the future, with continued research and technological advancements, we anticipate discovering more effective predictive methods to offer additional possibilities for early diagnosis and treatment of fetal growth restriction.

## Data Availability

The raw data supporting the conclusions of this article will be made available by the authors, without undue reservation.
